# Suppression of bacteriocin resistance using live, heterospecific competitors

**DOI:** 10.1111/eva.12797

**Published:** 2019-05-03

**Authors:** Amrita Bhattacharya, Alexander Stacy, Farrah Bashey

**Affiliations:** ^1^ Department of Biology Indiana University Bloomington Indiana

**Keywords:** alternative antimicrobials, antimicrobial resistance, bacteriocins, competition

## Abstract

Rapidly spreading antibiotic resistance has led to the need for novel alternatives and sustainable strategies for antimicrobial use. Bacteriocins are a class of proteinaceous anticompetitor toxins under consideration as novel therapeutic agents. However, bacteriocins, like other antimicrobial agents, are susceptible to resistance evolution and will require the development of sustainable strategies to prevent or decelerate the evolution of resistance. Here, we conduct proof‐of‐concept experiments to test whether introducing a live, heterospecific competitor along with a bacteriocin dose can effectively suppress the emergence of bacteriocin resistance in vitro. Previous work with conventional chemotherapeutic agents suggests that competition between conspecific sensitive and resistant pathogenic cells can effectively suppress the emergence of resistance in pathogenic populations. However, the threshold of sensitive cells required for such competitive suppression of resistance may often be too high to maintain host health. Therefore, here we aim to ask whether the principle of competitive suppression can be effective if a heterospecific competitor is used. Our results show that a live competitor introduced in conjunction with low bacteriocin dose can effectively control resistance and suppress sensitive cells. Further, this efficacy can be matched by using a bacteriocin‐producing competitor without any additional bacteriocin. These results provide strong proof of concept for the effectiveness of competitive suppression using live, heterospecific competitors. Currently used probiotic strains or commensals may provide promising candidates for the therapeutic use of bacteriocin‐mediated competitive suppression.

## 1 INTRODUCTION

Rapidly spreading antibiotic resistance has created an urgent need to find novel antimicrobials (Allen, Trachsel, Looft, & Casey, 2014; Projan & Shlaes, 2004) and usage strategies that can constrain the emergence of resistance (Olofsson & Cars, 2007; Read, Day, & Huijben, 2011). One potential source of alternative antimicrobials is bacteriocins, which are proteinaceous toxins produced by bacteria, noted for their ability to kill closely related strains (Cotter, Ross, & Hill, 2013). Bacteriocins are ubiquitously produced by almost all known lineages of bacteria (Klaenhammer, 1988; Riley & Chavan, 2006), increasing their appeal as potential alternatives to replace antibiotics. However, resistance against bacteriocins has been frequently reported in natural as well as clinical isolates (Gordon, Riley, & Pinou, 1998; Hawlena, Bashey, Mendes‐Soares, & Lively, 2010; Koch et al., 2014). Relative to conventional antibiotics, little is known about the evolution of resistance against bacteriocins (Inglis, Scanlan, & Buckling, 2016). Here, we examine the evolution of resistance against bacteriocins in the insect‐pathogenic bacteria *Xenorhabdus bovienii* and conduct in vitro experiments to investigate a novel strategy for preventing the emergence of resistance.

The traditional approach of aggressive chemotherapy used with conventional antimicrobials employs high doses with the goal of eliminating all pathogen cells including partially resistant mutants (Ehlrich, 1913). However, by eliminating all sensitive cells, this approach imposes strong selection favoring resistant mutants that may be present at the onset of infection or be acquired over the course of infection (Day & Read, 2016; Read et al., 2011). Recent studies have proposed a “moderate chemotherapy” or “containment” approach that is more effective at suppressing resistant mutants from spreading. Moderate chemotherapy employs lower antimicrobial doses than aggressive approaches and does not eliminate all sensitive pathogenic cells. Competition between the untreated sensitive cells and resistant mutants results in competitive suppression of resistance and thereby prevents the spread of resistant mutants (Hansen, Woods, & Read, 2017; Kouyos et al., 2014; de Roode, Culleton, Bell, & Read, 2004; Wale et al., 2017). However, the success of this approach depends critically on maintaining a threshold density of sensitive cells for competitive suppression of resistance to occur. If this threshold is too high for a host to tolerate without succumbing to the infection, moderate chemotherapy will be ineffective. Under such circumstances, aggressive approaches are recommended (Hansen et al., 2017), which can be problematic as they impose strong selection for resistance. Thus, for sustainable use of antimicrobials, it is necessary to find strategies that can simultaneously achieve two major goals: (a) effective suppression of pathogen cell densities and (b) prevention of resistance evolution.

Over the course of debates on the relative efficacy of aggressive versus moderate treatment strategies, competition between drug‐sensitive and drug‐resistant pathogenic cells has repeatedly surfaced as a key determinant of the emergence and spread of resistance (Colijn & Cohen, 2015; Day, Huijben, & Read, 2015; Huijben et al., 2010; de Roode et al., 2004). Although models specifically emphasize competition between conspecific drug‐sensitive and drug‐resistant cells, the conceptual framework that underpins the role of competition in constraining resistance spread should be applicable to any ecological competitor. In other words, any strong competitor, whether it is a conspecific sensitive strain or heterospecific neighbor, may impose similar constraints on the spread of resistant mutants of the target strain if the competitor itself is unaffected by the antimicrobial used.

Here, we examine whether the conceptual basis of competitive suppression of resistance can be extended to include heterospecific competitors. We examine the evolution of resistance against bacteriocins and conduct in vitro, proof‐of‐concept experiments to determine whether using a heterospecific competitor strain in conjunction with bacteriocin dose can constrain the spread of resistant mutants in the population while also driving down densities of bacteriocin‐susceptible pathogenic cells. We expect high bacteriocin doses to impose selection favoring resistant mutants. In contrast, we predict that incorporating a heterospecific competitor with the high bacteriocin dose will reduce the emergence of resistance as the additional competitive challenge will impede the spread of resistant mutants. We further predict that low bacteriocin doses will result in lower selection for resistance than high doses; however, low doses may not effectively suppress total cell densities. We predict that using a competitor with low bacteriocin doses will not only slow the evolution of resistance but will also suppress untreated sensitive cells resulting in a better overall outcome relative to using the low dose without a competitor. Additionally, we predict that using only a competitor that itself produces a low dose of the bacteriocin should be effective at reducing total and resistant cell densities of the focal, target strain. Finally, these predictions rely on the assumption that resistance imposes a cost that makes resistant mutants inferior competitors to their sensitive counterparts. Thus, to determine whether resistance is indeed costly in our experiments, we also compare the growth of resistant mutants derived from bacteriocin‐exposed populations with the growth of sensitive cells.

## 2 MATERIALS AND METHODS

### 2.1 Experimental design

To examine the effect of dose on the growth of sensitive cells and evolution of bacteriocin resistance, cultures of a bacteriocin‐sensitive strain, *X. bovienii,* were exposed to two different doses of bacteriocin (high and low) and a “no bacteriocin” control. The effect of competitive suppression was examined by either introducing a live competitor strain in conjunction with the bacteriocin dose (“high dose + competition” and “low dose + competition”) or exposing cells to bacteriocin alone. Finally, the effect of a bacteriocin‐producing competitor alone was examined by introducing a competitor that produces a low dose of the bacteriocin to the cultures of the sensitive strain (bacteriocin‐producing competitor). In all treatments, we determined both total and resistant cell densities of the focal, target strain *X. bovienii*.

### 2.2 Bacterial strains

A natural isolate of the bacteriocin‐sensitive strain *X. bovienii*, Bov59 (isolated as described in Hawlena, Bashey, Mendes‐Soares, et al., 2010), was used as the focal, target strain. Both high and low doses of bacteriocin were derived from a natural isolate of *Xenorhabdus koppenhoefferi,* Kop46 (isolated as described in Hawlena, Bashey, and Lively (2010)), which is also a sympatric competitor of the bacteriocin‐sensitive strain (Hawlena, Bashey, & Lively, 2012). The competitor used in the “high dose + competitor” and “low dose + competitor” treatment was a mutant *X. koppenhoefferi* strain (Kop46 mut) that does not release functional bacteriocin (construction as described in Morales‐Soto and Forst (2011)). For the “bacteriocin‐producing competitor” treatment, the wild‐type Kop46 strain was used. These strains of *X. koppenhoeferi* and *X. bovienii* are morphologically distinct whereby Kop46 colonies appear maroon and Bov59 colonies appear blue on NBTA plates (nutrient agar supplemented with 0.0025% [w⁄v] bromothymol blue [Sigma‐Aldrich] and 0.004% [w⁄v], 5 triphenyltetrazolium chloride [Sigma‐Aldrich], pH = 8). In addition, Bov59 has a higher natural resistance to ampicillin than Kop46, thereby enabling further distinction on NBTA plates with 75 μg/ml ampicillin. All cultures used in the experiment were derived from freezer stocks maintained at −80ºC and streaked onto NBTA plates prior to each replicate.

### 2.3 Bacteriocins

Bacteria in the genus *Xenorhabdus* produce a phage tail‐like bacteriocin, xenorhabdicin (Boemare, Boyer‐Giglio, Thaler, Akhurst, & Brehelin, 1992; Thaler, Baghdiguian, & Boemare, 1995). Like most bacteriocins, xenorhabdicin has a narrow‐killing range, affecting other *Xenorhabdus* strains and strains in the closely related genera *Photorhabdus* and *Proteus*. Xenorhabdicin is similar in structure to R‐type pyocins, where killing requires attachment of tail fibers to the target cell and results from a puncture and subsequent depolarization of the cell membrane (Williams, Gebhart, Martin, & Scholl, 2008). Xenorhabdicin is encoded by a remnant P2 phage cluster, approximately 30 kb in length (Ciezki, Murfin, Goodrich‐Blair, Stock, & Forst, 2017; Morales‐Soto & Forst, 2011; Morales‐Soto, Gaudriault, Ogier, Thappeta, & Forst, 2012). Growth inhibition of the target strain Bov59 by the xenorhabdicin produced by Kop46 was determined by insertional inactivation of the sheath gene and subsequent lack of inhibitory phenotype observed in the mutant phenotype (Bashey, Forst, and Palmer, unpublished data).

### 2.4 Bacteriocin doses

Both high and low doses of the bacteriocins used were procured from wild‐type Kop46 cultures. High‐dose bacteriocin extracts were collected from Kop46 cultures that were chemically induced with mitomycin C (Sigma‐Aldrich). Briefly, pure cultures of Kop46 cultures in exponential phase were incubated with 0.5 µg/ml mitomycin C. After overnight incubation at 28°C, bacteriocin extracts were collected by centrifuging cultures at 1620 G for 5 min and filtering the supernatant through 0.45‐µm filters (Acrodisc). This procedure allows the bacteriocin to pass through while eliminating any cells in the extract. Previous work with this isolate has shown that PEG precipitations of filtered supernatants (to further isolate the phage tail‐like bacteriocins) show identical patterns of inhibitory activity as the unprecipitated doses used here, when tested against over 10 different target genotypes. Further, no active phage has been found in the strains used in this study (Hawlena, Bashey, & Lively, 2010).

The low dose was obtained from stationary phase cultures of Kop46 without chemical induction. This choice of the low dose of bacteriocin was deliberate to ensure that the amount of bacteriocin produced by the competitor in the “bacteriocin‐producing competitor only” treatment matched the low dose used in the “low dose + competition” treatment. This is further confirmed by earlier work showing that the concentration of bacteriocin produced by Kop46 is not affected by coculturing with Bov59 (Bhattacharya, Pak, & Bashey, 2018). Finally, the inhibitory profile of Kop46 bacteriocin with and without chemical induction was identical in nine tested strains, indicating that the same antimicrobial is released at both doses used in the experiment. All bacteriocin extracts were stored at 4°C until used.

### 2.5 Bioassay to quantify high and low bacteriocin doses

A growth inhibition bioassay (previously described in Bhattacharya et al., 2018) was used to quantify and compare the high and low doses of bacteriocin used in the experiments. This bioassay compares the inhibitory effect of different bacteriocin doses by comparing the duration of lag imposed on the growth of a sensitive strain by fixed amounts of the bacteriocins. To do this, we mixed a bacteriocin dose (or culture media in the “no bacteriocin” control) and a starting culture of sensitive cells in 1:5 ratio by volume. The growth of the sensitive strain in the presence and absence of bacteriocin doses was measured on a Bioscreen optical plate reader (GrowthCurves). The doses examined in this experiment included the chemically induced “high dose,” three 10‐fold serial dilutions of the high dose, and the “low dose” derived from chemically uninduced Kop46 cultures. Four replicate wells of each bacteriocin dose were examined in a 100‐well plate reader (Honeycomb plate, Growth Curves). Each well contained 200 µl of the respective bacteriocin + culture mixture at an initial density of 10^6^ CFU/ml. Control wells with un‐inoculated media were included to rule out contamination. The plates were incubated in the optical plate reader at 28°C with continuous shaking at medium amplitude, and OD_600_ was recorded every 30 min for 24 hr.

**Figure 1:**
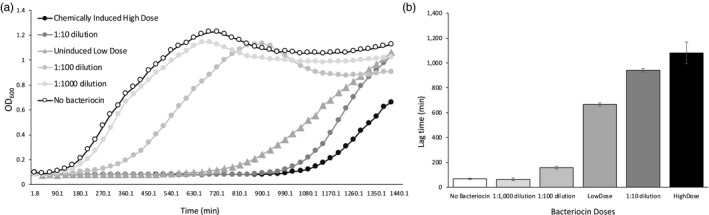
Bacteriocin doses measured by induced growth inhibition of sensitive strains. (a) Growth curves of a bacteriocin‐sensitive strain when exposed to different bacteriocin doses. Doses include the chemically induced “high dose” used in this study, three consecutive 10‐fold dilutions of the high dose and the “low dose” which was derived from chemically uninduced, 24‐hr cultures of the bacteriocin‐producing strain. Each marker point represents an average OD_600_ value of four technical replicates. (b) Mean ± *SEM* lag times of the bacteriocin‐sensitive cultures exposed to the different bacteriocin doses. Exposure to higher bacteriocin doses results in increased lag times due to growth inhibition

The OD_600_ data were used to generate growth curves, and the lag time of the cultures growing in each well was calculated using the software GrowthRates 3.0 (Hall, Acar, Nandipati, & Barlow, 2013). Lag time represents the time taken by a starting culture to reach exponential growth phase. The inhibitory activity of bacteriocins results in increased lag times of cultures that are exposed to bacteriocins relative to negative control cultures that are not exposed to bacteriocins (Figure 1). This increase in lag time provides a metric of bacteriocin inhibitory activity and thus dose. A growth inhibition bioassay revealed that the lag times of Bov59 cultures exposed to the “high‐dose” bacteriocin were significantly longer than the lag times of cultures exposed to “low‐dose” bacteriocin (*F*_1,18_ = 62.41, *p* < 0.0001, Figure 1). The “low‐dose” bacteriocin imposed significantly greater growth inhibition than the “no bacteriocin” control (*F*_1,18_ = 130.64, *p* < 0.0001), 1:1,000 dilution (*F*_1,18_ = 132.25, *p* < 0.0001), 1:100 dilution (*F*_1,18_ = 94.09, *p* < 0.0001) and significantly less inhibition than the 1:10 dilution (*F*_1,18_ = 27.45, *p* < 0.0001) bacteriocins (Figure 1).

### 2.6 Experimental protocol to determine the effects of dose and competition

Starting cultures (~10^6^ CFU/ml) of bacteriocin‐sensitive Bov59 were established by inoculating 5‐ml LB media (Difco) with 100 µl of an overnight culture in 20‐ml culture tubes. To examine the effect of dose on the evolution of bacteriocin resistance, cultures were either exposed to 1 ml of bacteriocin dose (high or low) or 1 ml additional LB (“no bacteriocin” control). The effect of competitive suppression was examined by either introducing a live competitor strain (Kop46 mut) in conjunction with the bacteriocin dose or exposing cells to bacteriocin alone. The mutant Kop46 strain was co‐inoculated in the competition treatment tubes (50 µl of an overnight culture), in addition to the respective bacteriocin dose. Finally, the effect of a bacteriocin‐producing competitor alone was examined by directly co‐inoculating 50 µl of overnight cultures of a bacteriocin‐producing competitor (Kop46) without any additional bacteriocin dosage. All cultures were incubated for 24 hr at 28°C with shaking at 120 rpm (New Brunswick C2 platform shaker). Nine independent experimental replicates of all the treatments were performed.

After 24 hr, serial dilutions were plated to estimate total and resistant cell densities of the focal, target strain Bov59, as well as, densities of the heterospecific competitors. Total density of the Bov59 cells was estimated by plating 100 µl of the culture dilutions on NBTA agar plates with 75 μg/ml ampicillin (NBTA + Amp75). Both bacteriocin‐sensitive and bacteriocin‐resistant Bov59 cells can grow on these plates but the heterospecific competitor used in the experiment does not grow on these plates. Moreover, the Kop46/Kop46 mutant colonies are morphologically distinct from Bov59 allowing the colony counts of each species to be further confirmed.

To determine resistance, 100 µl aliquots of the dilutions were mixed with equal volumes of chemically induced bacteriocin before plating (as in Bashey, Young, Hawlena, & Lively, 2012) allowing only resistant colonies to grow. This method of measuring resistance evolution was independently validated as is described in the following section. Colony counts for total Bov59 and bacteriocin‐resistant Bov59 determined from these plates were used to calculate the proportion of resistant mutants in each treatment. Additionally, one dilution was plated on NBTA agar plates without ampicillin which is a nonselective media plate and thus confirmed the absence of contamination in cultures. In all cases where no colonies were detected on a plate, the detection limit of cell density for that plate was used instead of zero, to be as conservative with estimates as possible.

### 2.7 Growth inhibition bioassay to validate resistance evolution

To validate the method used for detecting resistance evolution, 10 colonies from one replicate of the “high‐dose” treatment were chosen at random. These 10 test colonies were derived from a NBTA + Amp75 plate used to calculate total Bov59 density. The colonies were picked, inoculated in 5 ml LB media overnight, and then used to conduct a growth inhibition bioassay as described above in the Bioassay section. All 10 test colonies, and a bacteriocin‐sensitive, wild‐type Bov59 culture were grown in the presence and absence of chemically induced bacteriocin. Starting cultures were mixed with bacteriocin or additional growth media in a 5:1 ratio, and growth of the cultures was measured in an optical plate reader. OD_600_ readings were taken every 30 min for 24 hr. The resulting growth curves were examined to look for bacteriocin‐mediated inhibition. Typically, sensitive cultures show strong bacteriocin‐mediated inhibition of growth depicted by increased lag times in the presence of bacteriocin relative to the absence of bacteriocin (as shown in Figure 1). Resistance was determined as the lack of strong inhibition in the presence of bacteriocin. Among the examined colonies, nine out of 10 showed resistance. The resistant colonies showed a significantly shorter lag time when exposed to bacteriocin (mean ± *SEM* = 42.62 ± 7.9 min) relative to the sensitive cultures (mean ± *SEM* = 1,263.71 ± 46.68 min, *t* = 25.79, *df* = 1, *p* = 0.02). The proportion of resistant colonies (nine out of 10, i.e., 90%) was consistent with the proportion calculated for that replicate (89.13%) by comparing Bov59 CFUs from plating with and without bacteriocin, thereby validating the experimental methods used to measure resistance evolution.

### 2.8 Cost of resistance

To determine whether the evolution of resistance against bacteriocins is associated with growth costs, growth parameters while growing in the absence of bacteriocin were compared for sensitive and resistant colonies isolated from two independent experiments. First, we examined the 10 test colonies derived from the current experiment described in the previous paragraph. Growth parameters in the absence of bacteriocin for the nine resistant colonies were compared to growth parameters of the sensitive colony and a wild‐type Bov59 colony. OD_600_ values for four replicate wells for each colony were measured by the Bioscreen optical plate reader and were used to estimate colony average growth rate and maximum OD values by the software GrowthRates 3.0 (Hall et al., 2013). These colony averages were used to compare growth between resistant and sensitive colonies.

Resistant colonies were also isolated from an independent experiment. In this second experiment, initial cultures of wild‐type Bov59 were grown in the presence (Exposed) and absence (Negative) of a high dose of bacteriocin in a 100‐well microtiter plate (Honeycomb plate, Growth Curves). Four independent replicates from each condition were pooled, and serial dilutions were plated on NBTA agar plates. Five separate colonies from each treatment (Exposed and Negative) were preserved by growing overnight in LB and making freezer stocks in glycerol, which were stored at −80ºC. The freezer stocks were used to conduct growth inhibition bioassay as described above. All five “Exposed” stocks showed resistance to bacteriocins, while all five “Negative” treatment stocks showed bacteriocin sensitivity. Growth parameters of these colonies in the absence of bacteriocin were determined as described in the previous paragraph.

### 2.9 Statistical analysis

The effect of different bacteriocin doses on the lag times of sensitive Bov59 cultures were compared by performing a one‐way analysis of variance using “bacteriocin dose” as a fixed effect in Proc Mixed. To compare the effect of bacteriocin dose and the presence of a competitor on log_10_‐transformed cell densities and proportion resistance values, mixed model analyses of variance with “bacteriocin dose” and “competition” as fixed effects and “experimental block” as random effect were used in Proc Mixed. Bacteriocin‐induced delay in lag time, growth rates, and maximum OD values of sensitive and resistance cultures were compared using Proc Ttest with the Satterthwaite approximation to account for unequal variance and sample sizes. To visualize growth of resistant and sensitive colonies, a LOESS regression on the colony average OD was used to estimate the mean OD per time for each type of colony grown. All analyses were performed in SAS 9.4.

## 3 RESULTS

### 3.1 Exposure to high bacteriocin dose reduces total Bov59 densities but imposes strong selection for resistance

**Figure 2:**
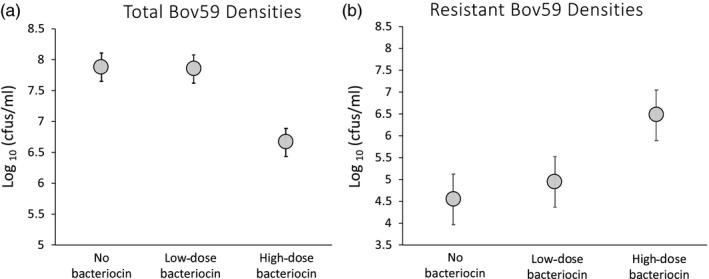
Effectiveness of bacteriocin dose (a) and evolution of resistance (b) in response to exposure to no, low, or high doses of bacteriocin. Total cell densities of the target strain Bov59 (a) are significantly lower in the high bacteriocin treatment as compared to low bacteriocin and negative control. However, the density of resistant Bov59 cells (b) of the target strain is significantly higher in the high bacteriocin treatment than other treatments. Data are shown as log_10_ (CFU/ml) after 24‐hr exposure. Gray circles represent the mean of nine experimental replicates with 95% confidence intervals. Detection limit for cell density counts was 10^5^ CFU/ml for the “low‐dose” and “no bacteriocin” treatments and 10^4^ CFU/ml for the “high‐dose” treatment

Exposure to a high dose of bacteriocin resulted in significantly lower total Bov59 cell densities (Figure 2a) than the “no bacteriocin” (*F*_1,16_ = 87.23, *p* < 0.0001) and “low‐dose bacteriocin” treatments (*F*_1,16 = _83.53, *p* < 0.0001). Specifically, the high‐dose treatment had over an order of magnitude reduction in total Bov59 densities (high‐dose CFU/ml = 6.6 × 10^6^ vs. 7.8 × 10^7 ^and 7.1 × 10^7^ CFU/ml in the “no bacteriocin,” “low‐dose” treatments, respectively). While this shows the effectiveness of the high dose in reducing total cell densities, exposure to the high dose of bacteriocin also imposed strong selection for bacteriocin resistance (Figure 2b). The resistant Bov59 density was 35‐ to 62‐fold higher in the “high‐dose” treatment (5.1 × 10^6^ CFU/ml) relative to the “no bacteriocin” (mean CFU/ml = 2.1 × 10^5^, *F*_1,16_ = 46.51, *p* = 0.0001) and “low‐dose” (mean CFU/ml = 5.1 × 10^5^, *F*_1,16_ = 15.52, *p* = 0.0012) treatments. Although the “low‐dose” treatment had twice as many resistant cells as the “no bacteriocin” treatment, this difference was not statistically significantly (*F*_1,16_ = 1.08, *p* = 0.31). Total Bov59 densities measured in the low bacteriocin treatment were also not significantly different from the no bacteriocin treatment (F_1,16_ = 0.05, *p* = 0.82). It was confirmed, however, that the low bacteriocin treatment showed inhibitory activity against the target strain, using a growth inhibition assay (Figure 1).

### 3.2 Incorporating a live, heterospecific competitor with bacteriocin dose reduces total and resistant cell densities

**Figure 3:**
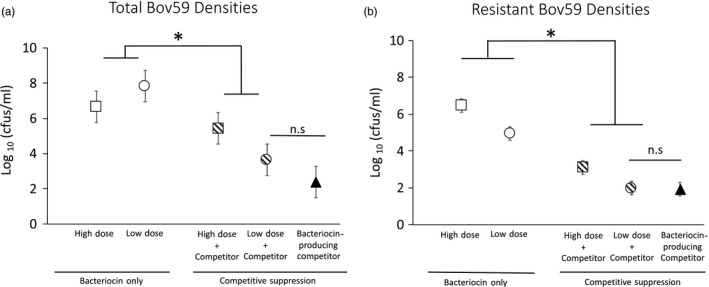
Suppression of total Bov59 density (a) and evolution of resistance (b) in response to bacteriocin exposure and competitive suppression. Only high and low doses of bacteriocin were administered in the bacteriocin only groups, while a live competitor was introduced in addition to bacteriocin doses in the competitive suppression group. The addition of a competitor significantly reduced the total cell densities (a) as well as resistant cell densities (b) of the target strain Bov59 at both doses of bacteriocin (*p < 0.0001). The combination of low dose and competitor was particularly effective at suppressing total cell densities (Dose x Competitor interaction: *F*_1,24_ = 13.8, *p* = 0.001), most likely due to the low levels of evolved resistance. Exposure to a bacteriocin‐producing competitor alone was as effective as the low dose with competition treatment (n.s., *p* > 0.05). Data are shown as log_10_ (CFU/ml) after 24‐hr exposure. Each symbol represents the mean of 9 experimental replicates with 95% confidence intervals for the respective treatment. Detection limit was 10^3^ CFU/ml for the “high dose + competitor” treatment and 100 CFU/ml for the “low dose + competitor” and “bacteriocin‐producing competitor” treatments

To examine the effectiveness of competitive suppression, a live competitor strain was introduced simultaneously with the bacteriocin doses. The addition of a competitor significantly reduced the total (*F*_1,24_ = 45.2, *p* < 0.0001) and resistant Bov59 densities (*F*_1,24_ = 254.7, *p* < 0.0001) relative to the use of bacteriocins only at both doses (Figure 3). Further, adding the Kop46 mutant competitor reduced the percentage of resistant cells in the high‐dose treatment from 83.6% to 0.3% (*F*_1,24_ = 128.14; *p* < 0.0001), demonstrating that competition prevented resistant mutants from spreading in the population. For the low‐dose treatment, percent resistance was 0.9% without the competitor. No resistant colony was detected in any of the nine replicates of the “low dose + competitor” treatment, at a detection limit of 100 CFU/ml, although total Bov59 density was on the order of ~10^5^ CFU/ml for this treatment. Overall, the “low dose + competitor” treatment yielded the lowest total and resistant cell densities in the target strain (Figure 3).

Despite the overall reduction in total cell densities upon the addition of a live, heterospecific competitor, the “high dose + competitor” treatment maintained a significantly higher density of Bov59 cells (mean CFU/ml = 1.8 × 10^6^) relative to the “low dose + competitor” treatment (mean CFU/ml = 6.5 × 10^3^, *F*_1,24_ = 01.04; *p* < 0.0042). Such high total densities of Bov59 in the “high dose + competition” treatment may reflect the escape of resistant mutants, despite a strong reduction in percent resistance upon the addition of the Kop46 mutant competitor. The density of resistant cells in the “high dose + competitor” treatment (mean CFU/ml = 1999.11) was significantly higher than the resistant cell density in the “low dose + competitor” treatment (mean CFU/ml = 99, *F*_1,24_ = 15.92; *p* = 0.0005). For replicates where no resistant colonies were found, the resistant cell density calculations used the detection limit instead of zero to be conservative. While no resistant colonies were detected in the “low dose + competition” treatment resulting in mean CFU/ml = 99 which was the detection limit, resistance was detected in one replicate of the “high dose + competition” treatment at a density of 10,000 CFU/ml suggesting that the likelihood of resistance escape was higher in the latter treatment. The mean density of the Kop46 mutant competitor itself was 0.9 × 10^6^ CFU/ml in the “high dose + competitor” treatment and 1.2 × 10^8^ CFU/ml in the “low dose + competitor” treatment.

### 3.3 Competition with bacteriocin‐producing competitor alone effectively suppresses total and resistant Bov59 densities

We examined whether using a bacteriocin‐producing strain, which releases bacteriocin matching the concentration of the low‐dose treatment, could be just as effective as the “low dose + competitor” treatment. Total and resistant cell densities of the target strain in this treatment (Figure 3) show that this approach is as successful as the low dose with competition treatment in suppressing target cells (*F*_1,8_ = 2.57, *p* = 0.15) and constraining resistance (*F*_1,8_ = 1.0, *p* = 0.35). No resistant colonies were detected in eight out of the nine replicates examined at a detection limit of 100 CFU/ml. The remaining replicate showed 0.5% resistance. The mean density of the Kop46 competitor itself was 1.2 × 10^8^ CFU/ml.

### 3.4 Resistant mutants pay a growth cost relative to sensitive cells

**Figure 4:**
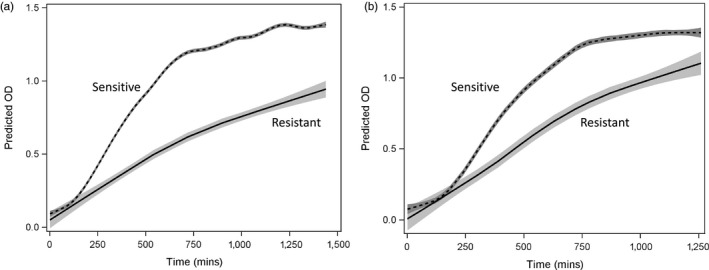
Cost of bacteriocin resistance as measured by reduced growth of resistant colonies (solid line, light gray shading) relative to sensitive colonies (dashed line, dark gray shading) when grown in the absence of bacteriocin. Predicted OD (±95% CI of mean) for two sensitive colonies and nine resistant colonies isolated from one replicate of the main experiment is shown in (a), while growth of five sensitive and five resistant colonies isolated from an independent experiment is shown in (b)

To determine whether resistant mutants pay a cost of resistance, we compared the growth in the absence of bacteriocin of resistant and sensitive colonies derived from two independent experiments. Nine resistant colonies from one “high‐dose” replicate of the main experiment were compared to the growth of the single sensitive culture from that replicate and a wild‐type Bov59 culture. Cultures derived from resistant colonies showed significantly reduced growth (Figure 4a, specifically they have lower growth rates (*t* = 3.87, *df* = 9, *p* = 0.003) and reach lower maximum OD values (*t* = 5.29, *df* = 9, *p* = 0.0004). Growth of five bacteriocin‐resistant and five bacteriocin‐sensitive cultures derived from a separate experiment also show distinct patterns in the absence of bacteriocin (Figure 4b). Again, consistent with a cost of resistance, bacteriocin‐resistant lineages show significantly reduced growth rates (*t* = 3.05, *df* = 4, *p* = 0.037) and a statistical trend toward lower maximum OD values (*t* = 2.48, *df* = 4, *p* = 0.06) relative to the bacteriocin‐sensitive lineages.

## 4 DISCUSSION

Conventional chemotherapeutic methods involving the use of high antibiotic doses may be effective initially, but as they impose strong selection favoring resistant mutants they ultimately result in treatment failure. Using lower doses can enable competitive suppression of resistance, when the host can tolerate susceptible pathogen loads that are sufficient to outcompete resistant mutants. However, using lower doses may not be feasible when the host cannot tolerate sufficient susceptible pathogenic cells to compete with resistant mutants. Here, we conduct in vitro experiments to test whether the principle of competitive suppression can be employed by using a heterospecific competitor in conjunction with antimicrobial dose to suppress resistance evolution, and overall pathogen load. We test this concept using bacteriocins and natural isolates of bacteriocin‐producing *Xenorhabdus* populations. Our results suggest that incorporating a live competitor strain in conjunction with bacteriocin can significantly reduce the density of resistant cells. We find that the combination of low‐dose bacteriocin and a heterospecific competitor is most effective at suppressing overall cell densities and the evolution of resistance against bacteriocins (Figure 3). We demonstrate that the same efficacy may be achieved by using a bacteriocin‐producing competitor strain which releases a low dose of the bacteriocin and simultaneously competes with the susceptible population. Finally, we find that the evolution of resistance against bacteriocins in vitro is associated with significant growth costs detectable in the growth rates as well as yield in cultures of resistant lineages relative to sensitive lineages (Figure 4). These results provide strong proof of concept for the effectiveness of employing competitive suppression to control the spread of bacteriocin resistance while also lowering susceptible cell densities.

This study examines the evolution of resistance against bacteriocins, which are anticompetitor toxins ubiquitously produced by bacteria. Bacteriocins have been proposed as alternatives to replace the fast‐depleting pool of antibiotics. In addition to being noted for their efficacy against pathogenic strains both in vitro (Noll, Sinko, & Chikindas, 2011; Sandiford & Upton, 2012) and in vivo (Goldstein, Wei, Greenberg, & Novick, 1998; Kruszewska et al., 2004; De Kwaadsteniet, Doeschate, & Dicks, 2009), bacteriocins demonstrate low cytotoxicity to host cells (Jasniewski, Cailliez‐Grimal, Chevalot, Millière, & Revol‐Junelles, 2009; Maher & McClean, 2006), contributing to their suitability as therapeutics (reviewed in Cotter et al., 2013; Riley et al., 2012). Bacteriocins typically have narrow‐killing spectra, although some broad‐spectrum bacteriocins are known (Lim et al., 2016; McAuliffe et al., 1998; Riley & Chavan, 2006). In the search for commercially viable therapeutics, broad killing spectra may seem appealing due to the wide range of pathogens one agent can treat. However, the potential risk of disrupting commensal microbial community within the host, and the increased potential for the evolution of resistance against the agent, may be important reasons to make narrow spectrum bacteriocins the favorable choice for sustainable therapeutic application (Rea et al., 2011). Further, the narrow‐killing range of bacteriocins may make them more amenable to the reduction of resistance evolution via the competitive suppression mechanism explored in our study as a resistant competitor strain is required. Although bacteriocins have been less well exploited as therapeutics, bacteriocin production is known to play a key role in the efficacy of probiotic supplements. Many probiotic bacteria confer health benefits via bacteriocin‐mediated competition resulting in their establishment in the microbiome (as reviewed by (Dobson, Cotter, Ross, & Hill, 2012; Gillor, Etizon, & Riley, 2009). Additionally, some studies have found strong evidence to suggest that bacteriocin‐producing probiotic strains can effectively inhibit pathogenic strains including enterohemorrhagic *Escherichia coli* and *Listeria monocytogenes* (Su, Henriksson, & Mitchell, 2007b, 2007a), further highlighting the potential for using bacteriocins as alternative therapeutics to replace antibiotics.

However, this potential may be squandered if bacteriocins are used in a conventional chemotherapeutic approach. Our results demonstrate that exposure to high doses of bacteriocin can lead to rapid evolution of resistance (Figure 2). In contrast, using a heterospecific competitor in conjunction with a high bacteriocin dose can suppress the spread of resistant mutants (Figure 3). The percent of resistant cells in the target population when a heterospecific competitor is added drops to 0.3% as compared to 87.6% in the presence of high dose without a heterospecific competitor. This striking reduction in the proportion of resistant cells when a competitor is present suggests that the heterospecific competitor was disproportionately constraining the spread of resistant mutants relative to sensitive cells. This observation is consistent with the hypothesis that resistant mutants pay costs of resistance that make them inferior competitors to their sensitive counterparts. We examined whether resistant mutants derived from exposure to high bacteriocin doses show growth costs. Across two independent experiments, we find that resistant cultures show significantly lower growth rates than sensitive cultures and achieve significantly lower optical densities after 24 hr of growth (Figure 4).

We find that a low bacteriocin dose did not impose strong selection for resistance, but was less effective at suppressing total densities of the target strain than the high dose (Figure 2). However, we find that incorporating a heterospecific competitor with low bacteriocin dose can allow the use of a competitor strain can allow low bacteriocin dosage to effectively reduce target cell density and significantly reduces the emergence of bacteriocin resistance. Further, a bacteriocin‐producing competitor used alone can be just as effective. These results provide promising leads to suggest that using a biotherapeutic agent such as a live, heterospecific competitor may confer increased sustainability to the use of bacteriocins as alternative therapeutics. The choice of the live competitor will be the most important, and perhaps, also the most limiting factor in the application of this therapeutic approach, as the competitor cells are expected to increase in density. Currently, in‐use probiotic strains may be the most viable candidates and could be further engineered to attack specific targets (reviewed in Sola‐Oladokun, Culligan, & Sleator, 2017; Williams et al., 2008). Additional candidates may be found by screening commensal bacterial strains for specific bacteriocin activity (Lakshminarayanan et al., 2013), as pathogenic strains often have nonpathogenic relatives. For example, the commensal *E. coli* G3/10 produces a bacteriocin that can inhibit enteropathogenic *E. coli* E2348/69 and has been effectively used in the treatment of irritable bowel syndrome (Zschüttig et al., 2012).

In summary, we have examined whether the concept of competitive suppression of resistance can be harnessed by incorporating heterospecific competitors in antimicrobial treatment. Our in vitro results suggest that the evolution of bacteriocin resistance can be significantly suppressed by either incorporating a live, nonbacteriocin‐producing competitor strain along with low bacteriocin dose, or using a bacteriocin‐producing agent alone. Both these treatments also successfully suppress the overall density of target cells regardless of resistance phenotype. Our experiment provides proof of concept for the use of biotherapeutic agents as a template for future investigations. Conceptually, this approach should be applicable across other antimicrobials as well and may be highly effective for treating a range of infections such as epithelial infections, including burns and wounds of the skin and mouth, as well as, gastrointestinal and lung infections, which can be recalcitrant to current therapies in immunocompromised, elderly, and cystic fibrosis patients. Future work investigating the efficacy of competitive suppression in spatially structured environments and in vivo models will lead to a greater understanding of the applicability of competitive suppression in treatment. In conclusion, we have provided proof of concept for a novel approach to impede the emergence of antimicrobial resistance and provide a template for future investigations to explore in relevant disease settings.

## CONFLICT OF INTEREST

The authors declare no conflict of interest.

## DATA AVAILABLITY

Data available from the Dryad Digital Repository: https://doi.org/10.5061/dryad.11kf1ck (Bhattacharya, Stacy, & Bashey, 2019).
